# Fairness-Based Tasks for Assessing Children’s Perceptions of Food Quantities and Associations with Portion Selection

**DOI:** 10.3390/nu10040453

**Published:** 2018-04-06

**Authors:** Aurore Ferrage, Lisa R. Fries, Nicolas Godinot, David Labbe, Nathalie Martin

**Affiliations:** Consumer Science & Applied Nutrition Department, Nestlé Research Center, 1000 Lausanne, Switzerland; aurore.ferrage@rdls.nestle.com (A.F.); lisa.fries@rdls.nestle.com (L.R.F.); Nicolas.Godinot@alimentarium.org (N.G.); david.labbe@rdls.nestle.com (D.L.)

**Keywords:** sensory, food design, portion size, children, food choice

## Abstract

It is critical to develop ecologically valid experimental methods to assess consumers’ food-related behaviors. Ad libitum approaches are often used but may not be appropriate for studies with children or with products that are not typically consumed until the individual feels full. The current study presents novel methods to assess children’s size perception and portion preference for gummy candies. In the first study, 62 children (30 boys, 32 girls) aged 6 to 9 years completed two matching tasks: one using pictures on a computer screen, and a similar task where the products were physically manipulated. Results of the two matching tasks were correlated, demonstrating that a computer-based approach could be used to predict the factors influencing children’s perception of food amount: the number, size, and shape of pieces. In the second study, a portioning measure was developed to investigate whether the factors identified in the matching tasks were confirmed in a task that more closely represented portion selection in the real world. The effects observed in the matching tasks could not be replicated in the portioning task. The size of each item had no significant impact on the portion selection, suggesting that it may be possible to reduce the size of pieces in snacks where multiple pieces are typically consumed without negatively impacting perceived quantity in children, thus offering a promising strategy to nudge children toward choosing smaller portions.

## 1. Introduction

Increased energy intake among children during the last three decades has been attributed to an increase in portion size as well as to the increase of the number of eating occasions per day [1]. One study found that snacking has increased to reach an average of three snacks per day, representing over 27% of the daily energy intake, with the largest increases in salty snacks and candy consumption [2].

A number of factors have been reported to impact children’s diet and energy intake, such as parental feeding practices [3,4,5,6], children’s food approach or food avoidance appetitive traits [7,8], children’s oral processing behaviors [9,10], and the sensory characteristics of the food that can influence the perception of quantities. Among the sensory cues that drive the eating experience, visual attributes seem to play a significant role on the amount of food selected and eaten [11]. The number and size of items [12,13] and the shape of the food [14,15] or the food container [16] affect the amount of snacks or beverage selected in adults and children.

Different methods exist to assess the factors influencing quantities of food selected and consumed. (For a recent review see Almiron-Roig et al. [17]) Laboratory-based food intake measurements are widely used to study eating behaviors. In these studies, participants are invited to eat as much or as little as they want of the tested food or beverage until they feel satiated and/or satisfied. The quantity of food or drink consumed is recorded. However, knowing the aim of the experiment may create experimenter demand effects, meaning that research participants may change their behavior due to cues about what they think constitutes appropriate or desirable behavior. Therefore, the bogus taste test is a measure widely used to identify factors that influence food intake [18]. It disguises the true purpose of the study (measuring food intake) under a cover story that presents the test as an assessment of taste perceptions and liking of the food. This approach is also used with children, although the demand effect may be weaker than in adults [13]. In some cases, the authors did not give a cover story and just asked children to choose as much of the food/snack as they wanted [19,20]. The risk of these approaches is that children may overindulge themselves, especially young children who do not often have the opportunity to select their own portions in the home environment or when the test food is a well-liked snack. In these cases, children may take such large (or variable) amounts of food that the results may no longer be sensitive to the experimental factors under study, and providing an unlimited amount of these foods could be considered to be unethical. To address this issue, we designed a new task where the children would create a portion of an appealing snack for another child of the same age rather than for himself, allowing us to measure the child’s perception of an appropriate portion without the child consuming it.

One limitation of such an approach, like most laboratory test meal experiments, is that it is labor-intensive to run and requires producing a substantial amount of prototype products. For example, it may be desirable to test a variety of prototypes, varying in dimensions such as shape, size, or color. To test these prototypes, we initially developed a computer matching task adapted to the cognitive ability of children from 6 to 8 years old [15]. The matching task is based on the concept of fairness that children understand from a young age and that leads school-aged children to generally be motivated to distribute resources fairly [21]. In this task, children were asked to divide portions between two cartoon characters. This projective technique was selected rather than having the children create portions for themselves and a friend, to avoid influences of greed or generosity, as well as to remove the expectation that the child would receive the portions of candy to eat. Within study 1, this computer-based task was compared to children’s responses on a similar matching task in which products could be physically manipulated, to assess the predictive power of the computer task. In study 2, children completed a portioning task, which was developed to reflect how children select portions at home. In this task, the child was asked to prepare a portion for a hypothetical other child. This approach was selected rather than asking the child to select her own ideal portion size to overcome potential tendencies of children to overindulge themselves when allowed to select their own portion of a well-liked product.

In study 1, we tested the hypothesis that a matching task performed on a computer reflects a matching task performed when real samples are manipulated. In study 2, we hypothesized that the product features identified as influencing portion selection in the matching tasks would also be identified as key factors predicting the size of the portions children would prepare in a more ecological portioning task.

## 2. Material and Method

### 2.1. Participants

Seventy-two children, aged 6 to 9 years, and a parent were recruited to participate in the study at three locations in England: Bourneville, Ware, and Staines. None of the children had any known food allergy or intolerance, or any food restrictions due to personal beliefs. All children were reported to like eating and have eaten gummy candies in the past three months. Children used a computer at least once a month. Written informed consent was obtained from all parents, and verbal assent was collected from children at the beginning of the study. Families were compensated for their participation, and children received a small bag of candy. The protocol was submitted to the UK Health Research Authority (HRA) to enter the ethical review process. On the basis of the application and confirmation that the research participants would be healthy volunteers, the HRA concluded that the study fell into the framework of a market research study, sourcing volunteers from a market research database, and therefore a review by the Research Ethics Committee within the HRA was not required. The study was then carried out in accordance with the Market Research Society code of conduct.

Six children participated in pilot sessions, two did not meet inclusion criteria (age), one was reported to be color-blind, and one withdrew during the course of the study. Data from the remaining 62 children (30 boys, 32 girls) with a mean age of 7.5 years (standard deviation = 0.14) were included in the analyses.

### 2.2. Stimuli

Bear-shaped gummy candies were selected as a reference product (REFERENCE gummy candy), and five other prototypes were produced. These were modified in shape through elongation (TALLER gummy candy) or flattening (WIDER gummy candy) displaying two different elongation ratios (height/width) compared to the reference. These changes were made either when keeping their depth (9 mm) and weight constant (3.5 g) or combined with a 30% size reduction proportionally distributed across the height and width dimensions (Table 1). This level of reduction was selected because it was sufficient to be noticeably smaller but not so extreme that the elongated and flattened variants would no longer be recognized as a bear-shaped candy.

Each of the six gummy variants was presented in four different numbers: three, five, eight, and 12. These quantities were selected because they were within the range of typical consumption for this type of product. Only red gummy candies were used to study the impact of number, shape (elongation ratio) and size on perceived quantity using a 4 (number) × 2 (size) × 3 (shape) experimental design including 24 trials. Six additional trials were added to study the impact of color variety on perceived quantity, but no effect of color was found (previously reported in [15] and not further discussed here).

### 2.3. Study 1: Matching Task

#### 2.3.1. Principle

Two matching tasks were developed to investigate the impact of number, shape and size on portion size perception. The tasks were presented as a game in which the child was asked to make fair piles of candy for two cartoon characters.

In both matching tasks, each of the six gummy variants was presented as test “piles” on the left of a PC screen or a two-plate display. For each test pile, the participant were instructed to add REFERENCE red gummy candies to the “selection” pile on the right to make the two piles fair. To facilitate the understanding of the notion of fairness and to familiarize the children with the task, training trials were done with guidance from the moderator before starting the real trials. Orange gummy candies were used for the training trials to illustrate that different numbers (two and seven reference candies), sizes, and shapes (an extra-large gummy candy of 29 mm height and 19 mm width) could be presented on the left part of the screen. For this trial with the large bear on the test pile, the experimenter explained that sometimes the candies on the left would be of different shapes and sizes, so the cartoon character would need more help to make the two piles fair. The experimenter continued to explain that if the candy on the left is bigger, the cartoon character might need more small bears to make it fair. For all trials, the child was told that at the end of the task, one of the cartoon characters would always choose first and would choose the biggest portion (left or right), leaving the other pile for the second character. Therefore, it was important that the children made the piles fair so that both characters would get a fair portion.

Each child completed two matching tasks, one with physical product manipulation (PPM) and the other with digital image selection (DIS), with task order counterbalanced across participants. The two tasks were performed during two separate sessions, scheduled one week apart, on the same day of the week at the same time.

#### 2.3.2. Physical Product Manipulation (PPM)

**Figure 1:**
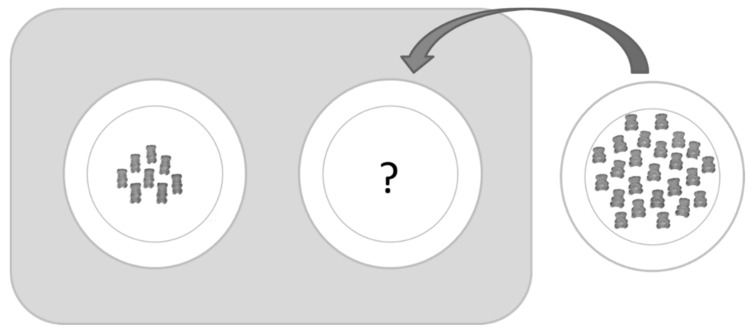
Illustration of the matching task in the physical product manipulation (PPM) condition: test pile on the left, selected pile of REFERENCE gummy candies on the right.

For each of the 30 trials, two plates were given to the child: the plate on the left contained a portion of test gummy variants, while the plate on the right was empty (Figure 1). A bowl of 24 REFERENCE gummy candies was provided to the child with the instruction to put these candies on the empty plate in an amount that would make the two plates into fair portions. The child could manipulate the gummy candies on the left plate, but had to put them back on the plate and could not eat them or move them to the plate on the right. The 30 different trials were presented in randomized orders, balancing position and order effect over all children.

#### 2.3.3. Digital Image Selection (DIS)

**Figure 2:**
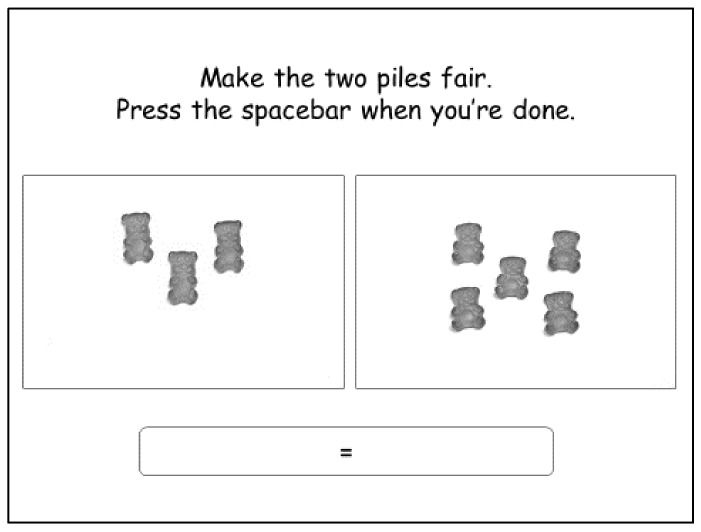
Screen capture of the matching task in the digital image selection (DIS) condition: test pile on the left, selected pile of REFERENCE gummy candies on the right.

A task mimicking the PPM task was developed on computer using the E-Prime 2.0 software (Psychology Software Tools, Pittsburgh, PA, USA). Each of the 30 test “piles” used in the PPM matching task were displayed on a white piece of paper and photographed with a high-resolution digital camera. The set-up for the photography kept the lighting, viewing angle and camera focus constant across photos. These 30 photographs, displayed as 642 × 384 pixels images on 1366 × 768 (HD) 14″ screens, were presented as the left (“test”) pile, following a randomized order automatically generated by the software. For each test pile, the participant were instructed to add between 1 to 24 REFERENCE gummy candies to the “selection” pile on the right to make the two piles fair. Each of the 24 incremental images of standard gummy candies were photographed following the protocol explained above. Participants selected the appropriate number of REFERENCE gummy candies by pushing the up arrow to add a candy to the pile, or the down arrow to take one away. When the piles were perceived to be fair, participants were instructed to press the spacebar to display the next trial (Figure 2). The items appeared in the same sequence of positions on the left and the right sides of the screen.

### 2.4. Study 2: Portioning Task

A portioning task was developed to measure the impact of number, shape, and size of the items on the size of the portion a child would make for another child. Children were asked to portion candies with the pretext of preparing a treat for hypothetical other children of the same age and gender coming the following day (e.g., “another six-year-old boy”). Eight gummy variants were presented to the children over two visits. For each gummy variant, a bowl of 24 gummy candies was placed in the center of the table and children were instructed to portion candies into a bag using their hands. Six bowls were prepared, each containing one of the prototype gummy candies described in Table 1. Two additional bowls composed of multicolored items of the reference shape and size (REFERENCE) were included in the experimental design, but the results will not be presented here. Bowl presentation order was randomized over children and split between the two sessions.

### 2.5. Data Analyses

#### 2.5.1. Matching Tasks

In each matching task, we measured the influence of the number, shape, and size of the gummy candies presented on the number of gummy candies selected with a fixed-factor 4 (unit number) × 3 (shape) × 2 (size) ANOVA. Interactions between variables of both ANOVAs were also explored. Post hoc paired comparisons were assessed by Fisher’s Least Significant Difference (LSD) test.

For the DIS condition, a stepwise linear regression was performed, with the number of units selected as dependent variables and the significant factors from the ANOVA as independent variables. The predicted number of candies selected for each gummy variant according to the model above was compared to the number of candies actually selected in the PPM task. The Pearson correlation coefficient (*R*^2^) was used for estimating the associations between these two sets of data and quantify to what extent the virtual computer (DIS) matching task could be used to predict the matching task including actual manipulation of the products (PPM).

#### 2.5.2. Portioning Task

We measured the influence of the shape and size of the gummy candies presented on the weight of the portion made with a fixed-factor 3 (shape) × 2 (size) ANOVA. Interactions between variables of both ANOVAs were also explored. Post hoc paired comparisons were assessed by Fisher’s Least Significant Difference (LSD) test.

The confidence level was set to 5% for all statistical analyses and data treatment was performed using IBM^®^ SPSS^®^ software version 21 (IBM Corporation, Armonk, NY, USA) and NCSS statistics software (NCSS, LLC, Kaysville, UT, USA).

## 3. Results

### 3.1. Matching Task: DIS *vs.* PPM

**Figure 3:**
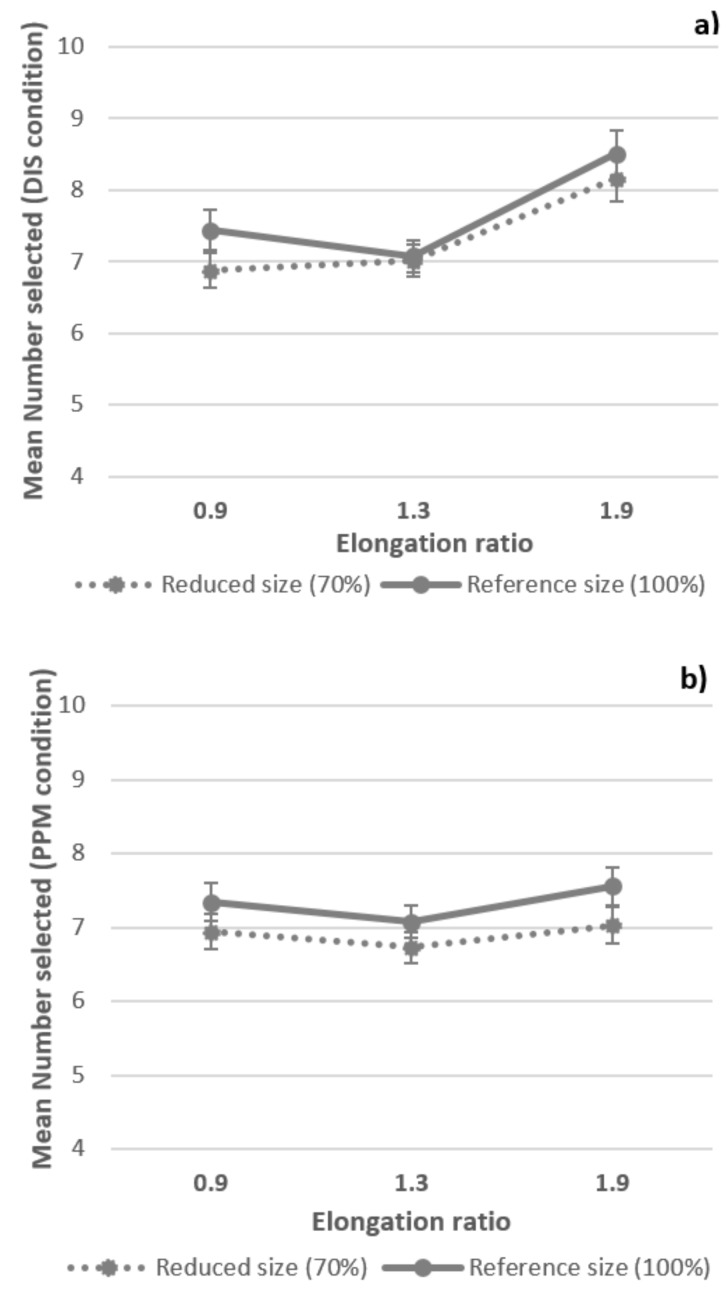
Mean number of gummy candies selected and standard error of the mean for the three elongation ratios (0.9; 1.3 and 1.9) and 2 sizes (reduced and reference) of candies for the DIS task (**a**) and PPM task (**b**).

A summary of the ANOVA results is presented in Table 2. The number of pieces presented had a significant effect on the number selected. Overall, children selected a number of reference gummy candies close to the number presented in the test pile. The size effect was also significant with fewer gummy candies selected when the reduced-size gummy candies were presented than for the full-sized ones. Finally, a main effect of shape on the number selected was also observed in both tasks but with differences in the LSD multiple comparison test (Figure 3). In the DIS task, significantly more pieces were selected to match the TALLER shape (ratio = 1.9) than for the WIDER (ratio = 0.9) or REFERENCE (ratio = 1.3) shape. In contrast, significantly fewer pieces were selected to match the REFERENCE shape than for the WIDER or TALLER shapes in the PPM task. Only one significant interaction was observed between number and shape in the DIS condition with a larger influence of elongation when a greater number of gummy candies presented. This effect did not reach significance in the PPM condition.

Based on the results of the ANOVA, a stepwise regression was conducted on the DIS data with the three following predictors: number of units, elongation, and size. The model resulting from the stepwise regression explains 72% of the variance in the number of units selected (*R*^2^ = 0.72; *F* = 1301; *p* < 0.001). The majority of the variance was explained by the number of gummy candies presented (partial *R*^2^ = 0.71; *F* = 3618; *p* < 0.001, beta = 1.07), with the elongation and size variables increasing the explained variance marginally, but significantly, by 1.42% (beta = 1.25) and 0.15 % (beta = 0.01), respectively.

**Figure 4:**
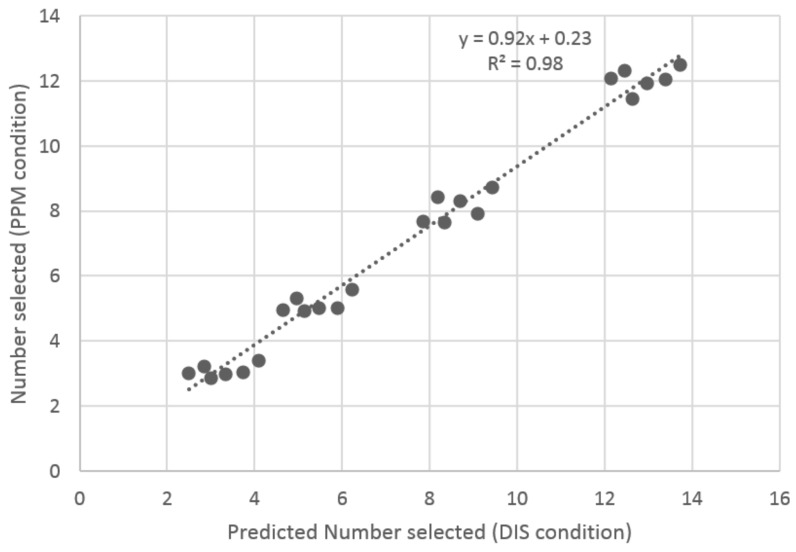
Scatterplots showing the relationship between the numbers of reference gummy candies selected in the PPM task and predicted from the DIS task for the 24 trials.

This model based on the DIS data was used to predict the number of gummy candies that we would expect to be selected for all 24 trial conditions in the PPM condition. There was a high correlation (*R*^2^ = 0.98, *p* < 0.001) (Figure 4) between the predicted data from the DIS matching task and the actual data of the PPM condition, suggesting that the results of the matching task performed on computer (DIS condition) were comparable to those obtained in the physical product condition (PPM condition).

### 3.2. Portioning Task

None of the variables, whether it was the size (*F*(1, 366) = 2.24, *p* = 0.14), the shape (*F*(2, 366) = 0.83, *p* = 0.44) of the gummy candies presented, or their interaction (*F*(2, 366) = 0.47, *p* = 0.62), had a significant impact on the number of gummy candies portioned. The type of prototype presented had no significant effect on the number of gummy candies portioned.

**Figure 5:**
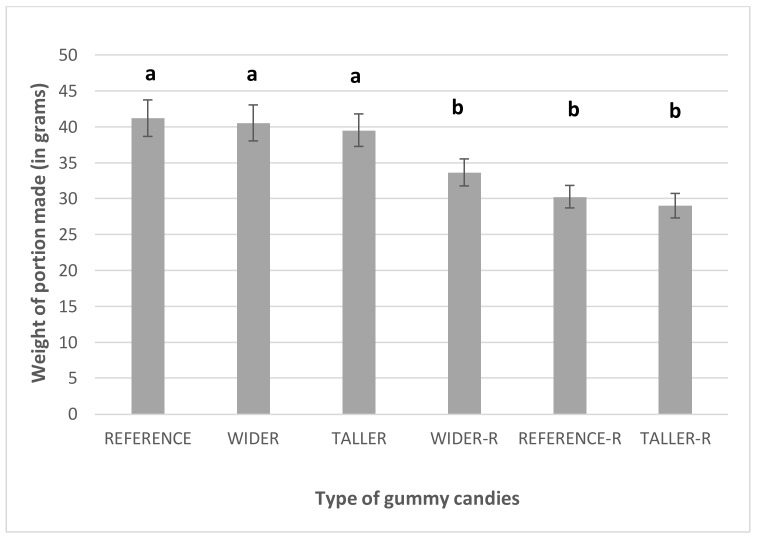
Mean and standard error of the mean of the weight of the portions made with the different types of gummy candies. Different letters represent a significant difference between samples according to the LSD multiple comparison test. – R added to the sample name accounts for the 70% reduced size.

The size (*F*(1, 366) = 29.94, *p* < 0.001) of the gummy candies was the only variable with a significant impact on the weight of the portions made. Since all portions were made with an approximately equivalent number of candies, the portions with candies reduced in size by 30% weighed less than the ones made with regular sized pieces (Figure 5).

## 4. Discussion

To our knowledge, this is the first time that fairness-based matching tasks have been used to assess children’s perception of quantity. The tasks were easy for the children to perform, likely due, at least in part, to the game-like structure of the task. The DIS computer task was a good predictor of the PPM task where the products could be physically manipulated. Both tasks highlighted the same main effects of the number of units and the size of each unit on the quantity selected. However, although the elongation effect was significant in both tasks, the ranking of the different elongation levels differed between the computer and the real matching task. The results of the DIS task confirmed our hypothesis that elongation would increase quantity perception. However, PPM task results showed that not only the most elongated but also the least elongated shapes were evaluated as being worth more standard pieces than the reference shape. This may be due to a novelty effect that may have been more prone to develop in the real product task rather than on a computer screen, where children are more accustomed to see unusual things (e.g., cartoons). When the gummy candies were physically manipulated, both the elongated and the wider shapes may have been perceived as more valuable because they were novel compared to the familiar reference shape. Another consequence of these divergent elongation effects was that the significant interaction between number and elongation observed for DIS was not replicated in PPM.

Different studies have reported the use of computer based tasks to measure food portion size related perceptions such as expected satiety [22], perceived volume [23], ideal portion size [24], portion size estimates [25], and perceived healthfulness [26], but few studies have investigated the predictive power of computer based tasks on actual behaviors and with mixed results. Wilkinson et al. [27] reported that a screen-based measure of expected satiety was a good predictor of ideal portion size assessed in a computer task and of eating behaviors associated with real food, i.e., ad libitum food intake measurement. Their conclusion is based on a high correlation between the computer virtual measures and the physical self-selected and eaten portions of a real meal based on only one food, a dish of pasta. Robinson et al. [28] showed through computer-based measures that screen exposure to larger portions increased the selected ideal portion size. However, they did not confirm this effect when participants actually served themselves of the real food and ate it, but again this was tested on only one food, potato chips. It is difficult to conclude from our study and the existing literature about the overall predictive power of computerized tasks, since this may depend on different factors. The contextual elements at play in the real situation may impact the decision making process of physically selecting and eating the food. For example, in a real consumption context where the candies are manipulated and can fall in other orientations, the elongation effect may be attenuated compared to presenting the gummy candies vertically on the computer screen which might have enhanced the salience of the elongation effect. Further, other factors, such as sensory-specific satiety might cause children to stop eating gummy candies, especially when, as in our study, they are all of the same flavor. It is also possible that the relative strength of the observed effects (i.e., the dominant effect of number) may also be largely responsible for the degree of reproducibility between computer and real tasks in the current study.

**Figure 6:**
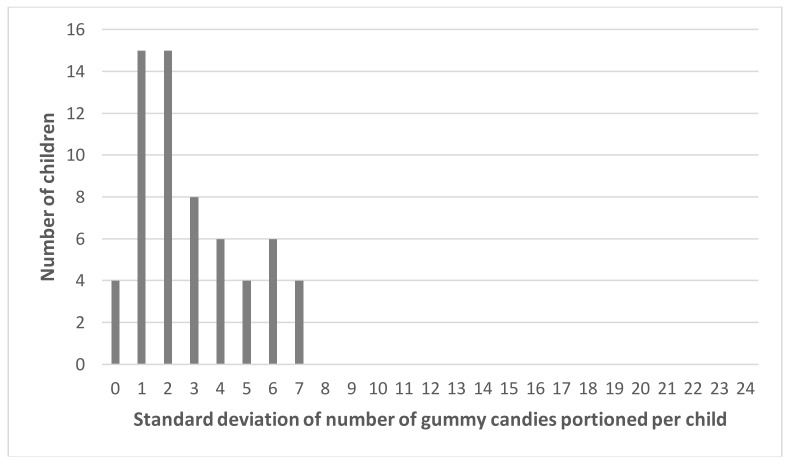
Distribution of standard deviations of the number of gummy candies portioned by each child in the different shape and size conditions.

**Figure 7:**
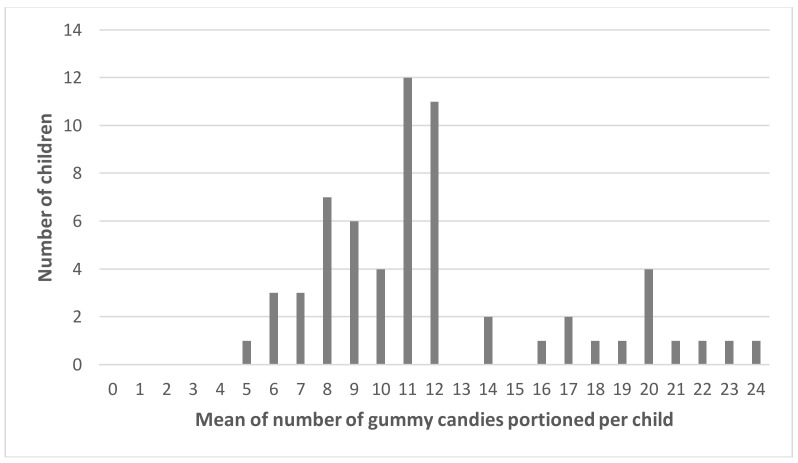
Distribution of means of the number of gummy candies portioned by each child in the different shape and size conditions.

The effects observed in the matching tasks could not be replicated in the portioning task. One possible explanation is that the matching task was more sensitive because it is based on a comparison process where attention is drawn to small difference such as a difference in elongation that may be difficult to detect when portioning. Another explanation could have been the large variability among children in the number of items portioned that could have overshadowed the differences in perception between variants for each child. To check for this, we calculated the standard deviation of the number of gummy candies portioned per child across the different conditions (different shapes and sizes from a fixed number available to portion). The distribution is positively skewed and peaks at a standard deviation between 1 and 2, highlighting indeed that most children portioned a similar number of gummy candies across conditions (Figure 6). Differences were greater between children than within a child. The distribution of the means of the number of gummy candies portioned per child is quite spread out and peaks at 11 and 12 (Figure 7).

The absence of effect of the size of each item is an important finding since it offers an interesting opportunity for item size reduction in multi-item snacks to nudge consumers, and especially children, to choose smaller portions while still being satisfied. This finding is consistent with previous work by Marchiori and colleagues that showed that cutting cookies in half reduced intake by 25% in children [13].

## 5. Conclusions and Recommendations

The present study shows that a computer task can deliver results that are highly correlated with a similar task where food stimuli are physically manipulated. However, although strong effects stand out in both matching tasks, they were not reproduced in a portioning task closer to the consumer reality. Consequently, we would recommend using the computer matching task developed in this study during a screening stage to identify the most promising effects or samples among a large range of options. This selection of samples would be better tested through a task that is as close as possible to the behavior of the target consumers in their everyday consumption context.

The portioning task results show that the size of each item did not have a significant impact on the number of pieces chosen to constitute the portion. This finding suggests that it is possible to reduce the size of pieces in snacks typically composed of several pieces without negatively impacting perceived quantity in children and thus offers an interesting strategy to nudge children toward choosing smaller portions.

## Figures and Tables

**Table 1:** Characteristics of the gummy candies used as stimuli.

Shape	Size	Height; Width (mm)	Elongation Ratio	Weight (g)
REFERENCE	Reference (100%)	25;19	1.3	3.5
TALLER	Reference (100%)	30;16	1.9	3.5
WIDER	Reference (100%)	21;23	0.9	3.5
REFERENCE	Reduced (70%)	21;16	1.3	2.5
TALLER	Reduced (70%)	25;13	1.9	2.5
WIDER	Reduced (70%)	17;19	0.9	2.5

**Table 2:** Results of ANOVAs for each of the matching tasks (DIS and PPM).

	DIS		PPM	
	Results of ANOVA	Results of Paired Comparison LSD Test	Results of ANOVA	Results of Paired Comparison LSD Test
Number of units	*F*(3, 1464) = 1302 ***	3 ^a^-5 ^b^-8 ^c^-12 ^d^	*F*(3, 1464) = 1875 ***	3 ^a^-5 ^b^-8 ^c^-12 ^d^
Size	*F*(1, 1464) = 8 **	Reduced ^a^-standard ^b^	*F*(1, 1464) = 23 ***	Reduced ^a^-standard ^b^
Shape	*F*(2, 1464) = 51 ***	0.9 ^a^-1.3 ^b^-1.9 ^b^	*F*(2, 1464) = 6 **	1.3 ^a^-0.9 ^b^-1.9 ^b^
Number × size	*F*(3, 1464) = 1.07, *p* = 0.35		*F*(3, 1464) = 1.49, *p* = 0.22	
Number × shape	*F*(6, 1464) = 4 **		*F*(6, 1464) = 0.41, *p* = 0.87	
Size × shape	*F*(2, 1464) = 1.54, *p* = 0.21		*F*(2, 1464) = 0.39, *p* = 0.68	

Values in the table are *F*-values with corresponding degrees of freedom for each of the simple effects and two way interactions with *, **, *** indicating significant effects at respectively 5%, 1%, 0.1%. In the Least Significant Difference (LSD) test, levels of each factors are ranked according to the test result, different subscripts (a, b, c, d) accounts for significant difference between each factor levels.
